# CLOCK Promotes Endothelial Damage by Inducing Autophagy through Reactive Oxygen Species

**DOI:** 10.1155/2016/9591482

**Published:** 2016-12-12

**Authors:** Xiao Tang, Changpo Lin, Daqiao Guo, Ruizhe Qian, Xiaobo Li, Zhenyu Shi, Jianjun Liu, Xu Li, Longhua Fan

**Affiliations:** ^1^Institute of Vascular Surgery, Department of Vascular Surgery, Zhongshan Hospital, Fudan University, Shanghai 200032, China; ^2^Department of Physiology and Pathophysiology, School of Basic Medical Sciences, Fudan University, Shanghai 200032, China; ^3^Department of Vascular Surgery, Qingpu Branch of Zhongshan Hospital, Fudan University, Shanghai 200032, China

## Abstract

A number of recent studies have implicated that autophagy was activated by reactive oxygen species (ROS). Our previous report indicated that CLOCK increased the accumulation of ROS under hypoxic conditions. In this study, we investigated the mechanisms by which CLOCK mediated endothelial damage, focusing on the involvement of oxidative damage and autophagy. Overexpression of CLOCK in human umbilical vein endothelial cells (HUVECs) showed inhibition of cell proliferation and higher autophagosome with an increased expression of Beclin1 and LC3-I/II under hypoxic conditions. In contrast, CLOCK silencing reversed these effects. Interestingly, pretreatment with 3-methyladenine (3-MA) resulted in the attenuation of CLOCK-induced cell autophagy and but did not influence the production of intracellular reactive oxygen species (ROS). Furthermore, Tiron (4,5-dihydroxy-1,3-benzene disulfonic acid-disodium salt), a ROS scavenger, significantly attenuated CLOCK-induced cell autophagy. In addition, we found that overexpression of CLOCK had no significant effects on the production of ROS and expression of Beclin1 and LC3-I/II under normoxic conditions in HUVEC. In this present investigation, our results suggested a novel mechanism of action of CLOCK in HUVECs, opening up the possibility of targeting CLOCK for the treatment of vascular diseases.

## 1. Introduction

Hypoxia has been associated with many cardiovascular diseases, including asphyxia, traumatic brain injury, hypertension, and varicose veins [1, 2]. Though intermittent hypoxia plays an important role in energy metabolism, angiogenesis, and vascular remodeling via the regulation of the hypoxia inducible factor-1*α* (HIF1*α*) [3, 4], continuous hypoxic microenvironment led to excessive release of reactive oxygen species (ROS), which accelerated protein oxidation, mitochondrial damage, cellular apoptosis, and necrosis [5, 6]. In normal physiological conditions, ROS could be eliminated by various enzymatic and nonenzymatic antioxidizing agents [7], while ROS accumulation results in oxidative damage and/or disruptive autophagy, which lead to aberrant mitochondrial function and cell injury by cellular stresses such as hypoxia, nutrient deprivation, and growth factor withdrawal [8–10]. A better understanding of the molecular mechanisms underlying the induction of autophagy by ROS may facilitate the development of new strategies for therapeutics.

The human Circadian Locomotor Output Cycle protein Kaput (CLOCK) belongs to the basic helix-loop-helix- (bHLH-) PER-ARNT-SIM (PAS) superfamily of transcription factors [11]. It was reported to play an essential role in regulating the expression of target gene expression by binding to E-box regulatory elements in target promoter regions [12]. Increasing evidence indicates that CLOCK functions as an oncogene at the cellular and molecular levels due to its involvement in cell proliferation, apoptosis, and DNA damage response [13, 14]. Our previous studies have shown that CLOCK induces Rho GTPase mediated endothelial dysfunction and NF-*κ*B mediated inflammatory responses via production of ROS [15]. Moreover, oxidative stress can be induced and initiate autophagy, which may contribute to endothelial dysfunction and tissue damage [16, 17]. Therefore, there is no doubt that investigating the molecular mechanisms of autophagy in vascular endothelial cells under hypoxia state may contribute to the development of new approaches to patients with vascular diseases.

Therefore, in the current study, we further investigated mechanisms of CLOCK in hypoxia-induced oxidative damage and autophagy in HUVECs. The results indicated that hypoxia induced HUVECs autophagy and apoptosis through upregulation of CLOCK which increased production of ROS.

## 2. Materials and Methods

### 2.1. Cell Culture and Treatments

HUVECs were obtained from ATCC (ATCC, Rockville, MD, USA) and cultured in EGM-2 Bulletkit medium (Lonza, Basel, Switzerland) as in our previous report [15]. The hypoxia model was performed as our previously described one using Xvivo Closed Incubation System (Xvivo system 300C, BioSpherix, Lacona, New York, USA) [15]. Tiron (4,5-dihydroxy-1,3-benzene disulfonic acid-disodium salt), a ROS scavenger, was purchased from Sigma-Aldrich (St. Louis, MO, USA) and dissolved in DMSO. Rapamycin and 3-methyladenine (3-MA) were obtained from Sigma and dissolved in DMSO.

### 2.2. Cell Viability Assay

Cell viability was determined by CCK-8 assay (Dojindo, Japan) following the procedures suggested by the manufacturer. In brief, approximately 2 × 10^3^ cells (in 100 *μ*L of fresh medium) were plated onto 96-well plates in triplicate, and the cells with different treatment were incubated with 10 *μ*L of the CCK-8 solution for an additional 2 h at 37°C. The optical density (OD) was measured at 450 nm using Infinite M200 microplate reader.

### 2.3. Apoptosis Assay

Cells were harvested and washed in ice-cold PBS. Then cells were incubated with Annexin V (PE Annexin V Apoptosis Detection Kit I, BD Biosciences, San Jose, CA, USA) for 15 min at 37°C in the dark and then incubated with propidium iodide (PI) for 10 min. The percentage of apoptotic cells was observed using the Elite ESP flow cytometer and data were analyzed with FlowJo 9.8.2 software.

### 2.4. Cell Migration Assay

Approximately 4 × 10^4^ cells (in 100 *μ*L serum free medium) were seeded on the upper chambers of a 24-well format cell culture insert with 8 *μ*m pores (Corning, Tewksbury, MA, USA). The lower chambers were filled with 600 *μ*L complete medium containing 20 ng/mL EGF (Sino Biological). After incubation at 37°C for 36 h, the migrating cells were fixed with 4% paraformaldehyde, stained with 0.5% crystal violet, and then counted in five randomly chosen microscope fields per filter under a light microscope (Olympus, Tokyo, Japan) at ×100 magnification.

### 2.5. Cellular ROS Level Analysis

Intracellular ROS was measured using the oxidant-sensing 2′,7′-dichlorofluorescein diacetate (DCFH-DA, 5 *μ*M, Invitrogen, Grand Island, NY, USA) as previously described [15].

### 2.6. Electron Microscopy

Cells were fixed with 2.5% glutaraldehyde at 4°C overnight. After removing the fixative, cells were then serially dehydrated in graded ethanol series, rinsed in propylene oxide, and incubated in propylene oxide and resin infiltration. Samples were embedded in Epon 812. The section was then visualized and photographed on Hitachi 7650 TEM (Hitachi, Japan).

### 2.7. Immunofluorescence and Confocal Microscopy

Cells were grown on coverslips in a 24-well plate overnight. Cyto-ID® Autophagy Detection Kit (Enzo Life Sciences, Plymouth Meeting, PA, USA) was used to detect the expression of LC3 according to the manufacturer's protocol. In brief, cells were seeded on coverslips in 6-well plates. Following different treatments, cells were suspended with 1x Assay buffer and stained with Cyto-ID Green Detection Reagent and Hoechst 33342 Nuclear from light for 30 minutes at 37°C. The pictures were captured using confocal laser scanning microscope (Nikon, Tokyo, Japan) and processed using the software provided by the manufacturer.

### 2.8. Western Blotting

Cells were lysed with ice-cold RIPA buffer (Cell Signaling Technology, Danvers, MA, USA), supplemented with a protease inhibitor cocktail (Sigma-Aldrich). Protein concentration was measured with a BCA protein assay kit (Thermo Scientific, Waltham, MA, USA). Equal amounts of extracts (40 *μ*g) were separated on 10% SDS-PAGE gels and then transferred onto PVDF membrane (Bio-Rad, Hercules, CA, USA). Membranes were blocked with blocking buffer (5% skim milk) for 2 h at room temperature and incubated with primary antibodies overnight at 4°C. The primary antibodies used are as follows: rabbit polyclonal LC3 antibody (Proteintech Group, Chicago, IL, USA), rabbit polyclonal HIF-1*α* antibody, Beclin1 antibody (Cell Signaling Technology), rabbit polyclonal CLOCK antibody, and beta-actin antibody (Abcam, Cambridge, MA, USA). After being washed in TBST, the membranes were incubated with horseradish peroxidase-conjugated secondary antibodies (Cwbiotech, China), and the protein bands were visualized using the SuperSignal™ West Pico Chemiluminescent Substrate (Thermo Scientific). Beta-actin was used as a loading control.

### 2.9. Statistical Analysis

The data were calculated and expressed as mean ± standard deviation (SD). All experiments were conducted at least three times. Differences between groups were evaluated using one-way ANOVA combined with Bonferroni's post hoc test and those between two groups were evaluated using Student's *t*-test. *P* values were considered significant when *P* < 0.05.

## 3. Results

### 3.1. Hypoxia Induced Autophagy and Repressed Cell Proliferation and Migration in HUVECs

**Figure 1:**
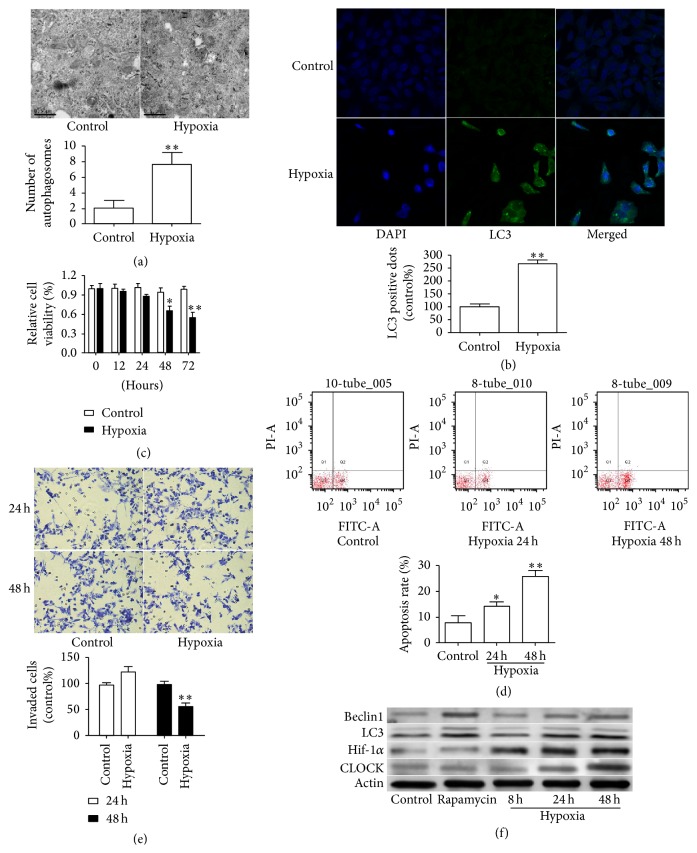
Effects of hypoxia on cell viability and autophagy in HUVECs. (a) Representative transmission electron micrographs showing autophagosomes in HUVECs at normoxia or hypoxia condition. Bars represent quantitative analysis. (b) The punctate GFP-LC3 dots were determined by confocal microscopy in HUVECs at normoxia or hypoxia condition. (c) CCK-8 assay was used to determine the proliferation of HUVECs as indicated treatment. (d) Flow cytometry detected the apoptosis of HUVECs for 24 and 48 h hypoxia. (e) Representative images of migrating cells for 24 and 48 h hypoxia. (f) The expression of CLOCK, HIF-1*α*, LC3, and Beclin1 was detected as indicated. Rapamycin was used as a positive control. The expression of *β*-actin was used as a loading control. ^*∗*^ *P* < 0.05, ^*∗∗*^ *P* < 0.01.

Previous studies showed that different degrees and durations of low oxygen could induce autophagy [9, 18]. To investigate whether autophagy was involved in HUVECs dysfunction under chronic hypoxia state, we first examined the procedure of autophagy. As the representative images showed in Figure 1(a), hypoxia increased the formation of autophagy bubbles in HUVECs under hypoxia (5% O_2_) for 48 h. Furthermore, the percentage of cells with punctate LC3 was also increased (Figure 1(b)). The effect of different durations of hypoxia on HUVECs viability was measured. Significant differences occurred at 48 h, though the cellular viability was observed to decrease at 24 h (Figure 1(c)). In addition, an increase in the percentage of cells apoptosis was observed following hypoxia at 24 and 48 h (Figure 1(d)). Then, we assessed mobility capability of HUVECs using migration assay under either normoxic or hypoxic conditions for 24 and 48 h in vitro, and the data showed that the amounts of cell migration induced by hypoxia for 48 h were significantly reduced compared with the migration value under normoxia (Figure 1(e)). Then, the expressions of LC3 and Beclin1, key components required for autophagy, were measured for further characterizing the autophagy under hypoxic state. As we expect, an increase in the level of CLOCK, HIF-1*α*, LC3-II, and Beclin1 was observed in HUVECs under hypoxia for 24 h and 48 h (Figure 1(f)), indicating that hypoxia induced cell damage and autophagy in HUVECs. We therefore selected 48 h as the time point to be used in the following experiments.

### 3.2. Inhibition of CLOCK Decreased Autophagy and Reversed Cell Dysfunction under Hypoxic Condition

**Figure 2:**
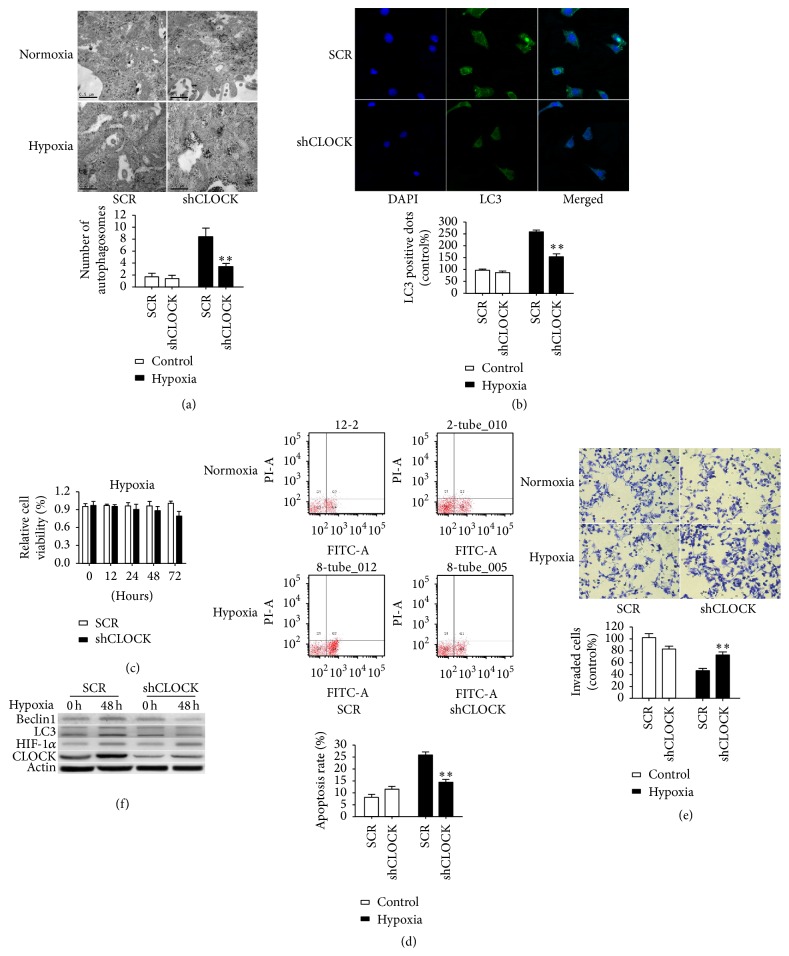
CLOCK silencing inhibited the effects induced by hypoxia in vitro. (a) HUVECs stably knock down CLOCK, followed by hypoxia treatment for 48 h. Autophagosome was determined by Electron Microscopy. (b) Representative fluorescence micrographs display the expression of LC3 in HUVECs with CLOCK knockdown under hypoxia condition. ((c)–(e)) Cell vitality, apoptosis, and migration capacity of HUVECs after CLOCK knockdown, followed by hypoxia treatment for 48 h. (f) Western blotting was used to detect CLOCK, HIF-1*α*, LC3, and Beclin1 expression. ^*∗∗*^ *P* < 0.01.

Our previous study has shown that CLOCK had played a crucial role in hypoxia-induced oxidative stress and cell damage [15]. To test whether CLOCK regulates autophagy under hypoxia condition, HUVECs stably knock down CLOCK cultured in complete medium at normoxia or hypoxia condition for 48 h. Interestingly, decreased autophagosomes were observed under hypoxia condition but there were no significant differences under normoxia condition (Figure 2(a)). CLOCK silencing resulted in a decrease of the percentage of HUVECs with punctate LC3 under hypoxia condition (Figure 2(b)), but there is no change at normoxia condition. Knockdown of CLOCK reversed hypoxia-induced inhibition of cell viability (Figure 2(c)), mobility (Figure 2(f)), and cell apoptosis (Figure 2(d)) under hypoxia condition, not under normoxia condition. Inhibition of autophagy by CLOCK silencing was also verified by western blot analysis that identified a significant decrease of LC3-II and Beclin1 expression in HUVECs exposure to hypoxia, compared to the negative control (SCR). However, the downregulation of LC3-II and Beclin1 expression was not significant under normoxia condition. Taken together, these results suggested that CLOCK was increased and involved in hypoxia induced autophagy and cellular functions under hypoxia condition.

### 3.3. CLOCK Aggravated Autophagy and Inhibited Cell Migration under Hypoxic Condition

**Figure 3:**
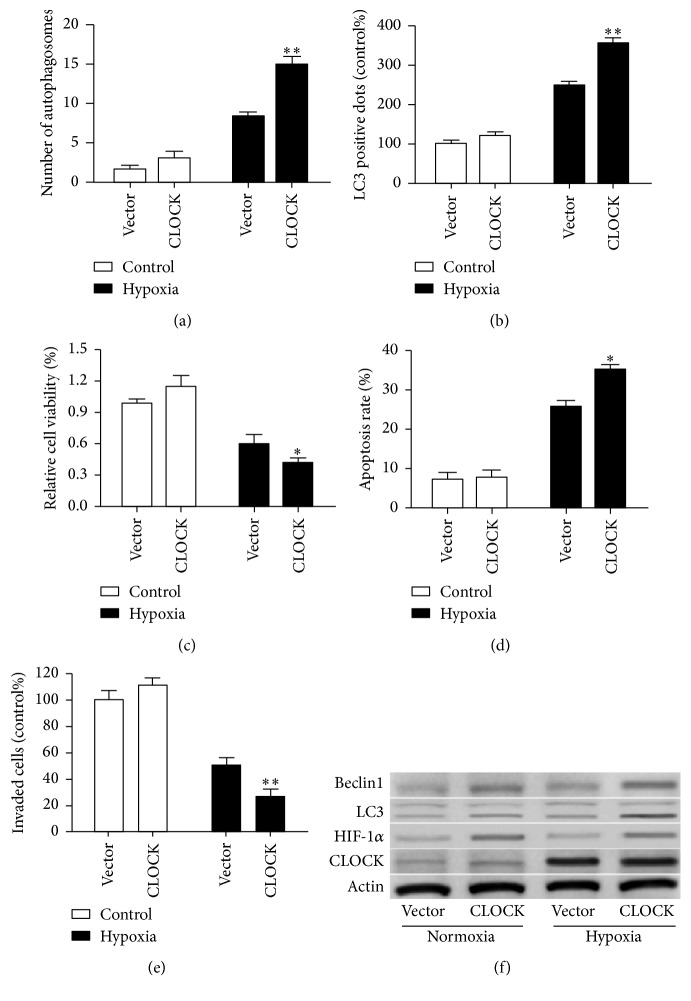
CLOCK overexpression aggravated HUVECs autophagy and caused cell damage. (a) HUVECs stable overexpression CLOCK was cultured in complete medium, followed by hypoxia treatment for 48 h. (b) Numbers of LC3-positive cells from three independent experiments were shown. (c) The function of CLOCK on cell vitality was assessed by CCK-8 assay. (d) The percent of apoptotic cells in CLOCK overexpressed HUVECs were counted by flow cytometry. (e) The migrating cells in CLOCK overexpressed HUVECs were counted. (f) The expression of CLOCK, HIF-1*α*, LC3, and Beclin1 was measured in CLOCK overexpressed HUVECs under normoxia or hypoxia condition. ^*∗*^ *P* < 0.05, ^*∗∗*^ *P* < 0.01.

In order to further confirm whether CLOCK regulated autophagy under hypoxia condition, HUVECs stable overexpression CLOCK was cultured in complete medium at normoxia or hypoxia condition for 48 h. Ectopically expressed CLOCK significantly increased the number of autophagosomes under hypoxia condition. Introduction of the CLOCK expression also enhanced the number of autophagosomes; however, the difference was not obvious under normoxia condition (Figure 3(a)). Furthermore, autophagic flux analysis of LC3-II expression confirmed this observation (Figure 3(b)). CCK-8 assay demonstrated that the proliferation of HUVECs significantly decreased in the CLOCK overexpression compared with the control vector under hypoxia condition (Figure 3(c)). Results from cell apoptosis and migration experiments show overexpression of CLOCK induced cell apoptosis (Figure 3(d)) and inhibited cell migration (Figure 3(e)) under hypoxia condition. However, cell viability, apoptosis, and capability of migration did not significantly change at normoxia condition. Moreover, western blot analysis showed that overexpression of CLOCK increased the expression of LC3-II and Beclin1 under hypoxia condition (Figure 3(f)). However, significant difference was not observed at normoxia condition (Figure 3(f)). These results suggest that CLOCK has played an important role in hypoxia-induced autophagy.

### 3.4. 3-MA Attenuates CLOCK-Mediated Autophagy and Migration under Hypoxic Condition

**Figure 4:**
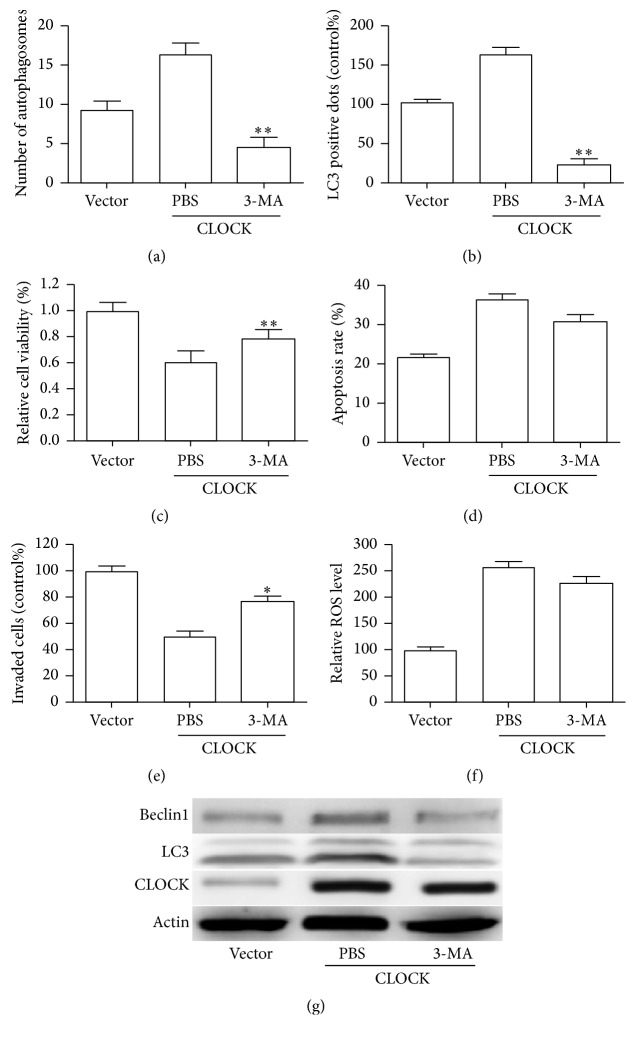
3-MA abolished the effects of CLOCK under hypoxia condition. CLOCK overexpressed HUVECs were pretreated with 3-MA (5 nM) for 2 h, followed by hypoxia treatment for 48 h. (a) Quantification of the number of autophagosomes. (b) The punctate GFP-LC3 dots were measured. (c) CCK-8 assay was used to assess the cell vitality. (d) Cell apoptosis was determined by a flow cytometry assay. (e) Cell migration was examined using transwell assays. (f) Bar graph showed the relative ROS level. (g) Immunoblotting for CLOCK, LC3, and Beclin1 expression in HUVECs as indicated treatment. ^*∗*^ *P* < 0.05, ^*∗∗*^ *P* < 0.01.

It has been reported that autophagy played a general role in coordinating growth and metabolism of endothelial cells [19–21]. To explore whether the CLOCK-induced inhibition of cellular function and increased LC3-II was owing to increased autophagy, HUVECs were treated with 3-MA (10 nM) to inhibit autophagic flux prior to hypoxia. As shown in Figures 4(a) and 4(b), compared with the PSB control, 3-MA treatment resulted in a decrease of number of autolysosomes and autophagy vacuoles. Then we examined the cell viability, apoptosis and capability of mobility. Our results have indicated that 3-MA treatment could obviously reverse CLOCK-induced inhibition of cell proliferation (Figure 4(c)) and mobility (Figure 4(e)), as well as cell apoptosis (Figure 4(d)). Autophagy could be enhanced by reactive oxygen species (ROS) in pathological conditions [22, 23]. Our previous study has shown that CLOCK mediated hypoxia induced production of ROS [15]. Then we assessed whether the production of ROS was changed in HUVECs treated with 3-MA. As shown in Figure 4(f), 3-MA treatment did not reverse the production of ROS induced by CLOCK. Moreover, autophagy markers including the LC3-II and Beclin1 were tested by western blotting. The expressions of LC3-II and Beclin1 were markedly decreased after 3-MA treatment (Figure 4(g)). These results demonstrated that autophagy was directly involved in CLOCK-mediated induction of cellular cytotoxicity.

### 3.5. ROS Is Involved in CLOCK-Induced Autophagy and Inhibition of Migration

**Figure 5:**
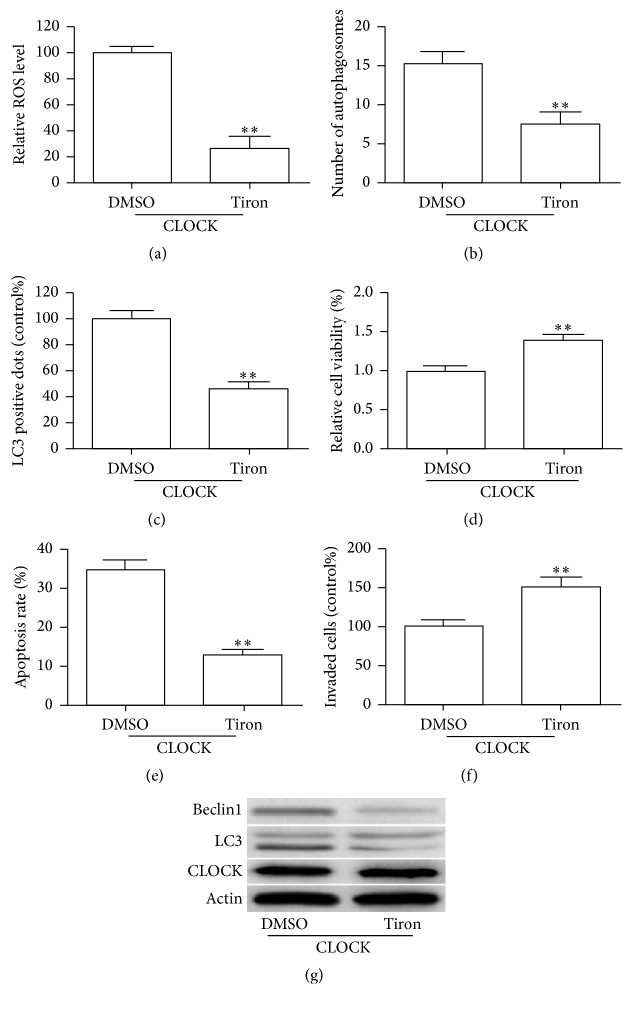
Involvement of ROS in CLOCK-induced cell autophagy and dysfunction under hypoxia condition. CLOCK overexpressed HUVECs were pretreated with Tiron (10 *μ*M) for 12 h, followed by hypoxia treatment for 48 h. (a) and (b) Bar graph quantifies the relative number of autophagosomes. (c) Quantification of LC3-II fluorescent-stained cells. (d) CCK-8 assay was performed to investigate the cell vitality. (e) Flow cytometry assay was performed to detect the cell apoptosis. (f) Transwell assay was used to assess the migration capacity. (g) Western blot analysis showing the expression of CLOCK, LC3, and Beclin1. ^*∗∗*^ *P* < 0.01.

Studies have shown that ROS played an important role in dysfunction of human vascular endothelial cells [24, 25]. Moreover, ROS was crucial in hypoxia-induced autophagy [26, 27]. Therefore, we further explored if ROS plays a role in CLOCK-induced cell autophagy and dysfunction in HUVECs. We eliminated the production of ROS with Tiron, a ROS scavenger (Figure 5(a)). Our results indicated that the stimulatory effects of CLOCK on the level of autophagy were significantly reversed by specific inhibition of ROS generation (Figures 5(b) and 5(c)). Furthermore, inhibition of ROS could abolish the adverse effect induced by CLOCK following hypoxia insult (Figures 5(d)–5(f)). In addition, western blot analysis showed that the levels of LC3-II and Beclin1 were decreased in Tiron-treated HUVEC cell in response to hypoxia (Figure 5(g)). These findings suggested that ROS played a critical role in CLOCK-induced cell autophagy and dysfunction.

## 4. Discussion

**Figure 6:**
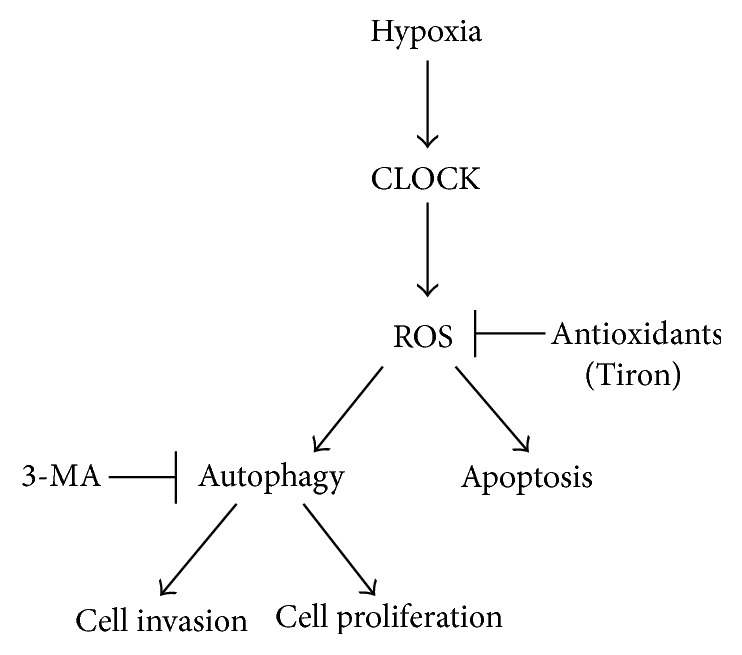
Schematic representation of CLOCK in hypoxia response. Hypoxia induced the expression of CLOCK, leading to ROS production in vascular endothelial cells. Increased cellular ROS resulted in excessive autophagy, which finally contributed to cell apoptosis and even cell death. Tiron, a ROS scavenger, decreased ROS production, which attenuated excessive autophagy.

In this study, we further investigated molecular mechanism of CLOCK in hypoxia-induced cell death. Previous studies have demonstrated that hypoxia caused injury via regulating autophagy [9, 27]. Consistent with these reports, our findings showed that hypoxia induced autophagy and endothelial dysfunction. CLOCK silencing attenuated the effects of hypoxia-induced autophagy and endothelial dysfunction. Antioxidants such as Tiron blocked ROS production thereby avoiding autophagy and cell damage. 3-MA also relieved cell damage by inhibiting autophagy, except cell apoptosis. A schematic illustration of the interaction model of hypoxia, CLOCK, ROS, and autophagy was shown in Figure 6.

Autophagy was highly conserved intracellular degradation of misfolded or aggregated cytoplasmic proteins, damaged organelles [10, 17]. Previous studies demonstrated that autophagy was involved in irreversible cellular dysfunction or cell death by various stressors, including ischemia, hypoxia, energy deprivation, and excitotoxic stimuli [10, 27]. By contrast, autophagy was reported to play a protective role in MMP-2-mediated cell transmigration and cell death in high glucose-stimulated HUVECs [28]. Autophagy can regulate high glucose-induced endothelial cellular senescence and apoptosis [29]. Therefore, the roles of autophagy in different condition may be beneficial, detrimental, or bifurcate. Autophagy was a protective factor for endothelial cells in the physiological state, which could scavenge the waste cytoplasmic cargo [30]. In our present study, the occurrence of autophagy was observed in HUVECs under hypoxia. Biological functions of HUVECs were also damaged response to hypoxia. The analysis of autophagy-associated proteins confirmed the induction of autophagy by hypoxia. Our previous study has shown that CLOCK was a positive regulator of ROS in HUVECs under hypoxia [15]. We further demonstrated that silencing of CLOCK expression can eliminate the effects of hypoxia on HUVECs. In contrast, overexpression of CLOCK triggered the levels of autophagy and the injury of HUVECs. There was no significant change under normoxia condition.

Since the relationship between ROS and autophagy has been investigated in many biological processes [7, 16, 19], we further explored whether ROS was involved in CLOCK-mediated autophagy. It was possible that autophagy at basal levels was required to maintain metabolism while excessive autophagic activation induced cell injury. In present study, we found that 3-MA, an inhibitor of autophagy, significantly inhibited cell autophagy and the protein levels of LC3-II and Beclin1. However, the intracellular ROS and apoptosis level were not changed. Furthermore, Tiron has been shown to diminish ROS production via removal of superoxide radicals [15, 31]. Therefore, we investigated whether ROS was involved in CLOCK-mediated activation of autophagy and endothelial cell injury. We found that Tiron could effetely suppress cell autophagy and restore cell viability. It has been shown that CLOCK was found to be significantly increased in gliomas and colorectal carcinoma [32, 33]. ROS levels were also increased in many cancers. So CLOCK-ROS might provide a new perspective for the clinical therapy. Therefore, our data indicated that dysregulation of ROS was crucial for CLOCK-stimulated cell autophagy and damage. However, other molecular mechanisms underlying continuous hypoxia-induced cell autophagy and damage remained to be elucidated.

In summary, the results of the present study indicated that hypoxia can induce autophagy in vascular endothelial cells through activation of the CLOCK-ROS pathway. It is possible to present a promising avenue for therapeutic opportunities for hypoxia-induced endothelial injury.
